# SNAI2/SLUG and estrogen receptor mRNA expression are inversely correlated and prognostic of patient outcome in metastatic non-small cell lung cancer

**DOI:** 10.1186/s12885-015-1310-1

**Published:** 2015-04-17

**Authors:** Akin Atmaca, Ralph W Wirtz, Dominique Werner, Kristina Steinmetz, Silke Claas, Wolfgang M Brueckl, Elke Jäger, Salah-Eddin Al-Batran

**Affiliations:** 1 Department of Hematology and Oncology, Krankenhaus Nordwest, UCT-University Cancer Center, Steinbacher Hohl 2-26, 60488 Frankfurt am Main, Germany; 2 STRATIFYER Molecular Pathology GmbH, Werthmannstraße 1, 50935 Cologne, Germany; 3 Institute of clinical research (IKF) at Krankenhaus Nordwest, UCT-University Cancer Center, Steinbacher Hohl 2-26, 60488 Frankfurt am Main, Germany; 4 Department of Internal Medicine 3, Klinikum Nürnberg, Prof.-Ernst-Nathan-Straße 1, 90419 Nuermberg, Germany

**Keywords:** SNAI2, SLUG, Estrogen receptor, NSCLC, Metastatic, Prognostic, Survival

## Abstract

**Background:**

Epithelial-mesenchymal transition (EMT) is involved in important malignant features of cancer cells, like invasion, metastatic potential, anti-apoptotic and stem-cell like phenotypes. Among several transcription factors, SNAI2/SLUG is supposed to play an essential role for EMT.

**Methods:**

Paraffin embedded tumor samples from 63 patients with metastatic non-small cell lung cancer, enrolled in a randomized phase II trial, were prospectively collected, 53 samples qualified for further analysis. Automated RNA extraction from paraffin and RT-quantitative PCR was used for evaluation of SNAI2/SLUG, estrogen receptor 1 (ESR1) and matrix-metalloproteinases (MMP) mRNA expression.

**Results:**

Clinical features like age, gender, performance status, histological subtype and stage were similarly distributed among SNAI2/SLUG positive and negative patients. SNAI2/SLUG was significantly, inversely correlated with ESR1 mRNA expression (p < 0.0001). In contrast, MMP2 (p = 0.387), MMP7 (p = 0.396) and MMP9 mRNA expression (p = 0.366) did not correlate with SNAI2/SLUG. Patients with high SNAI2/SLUG expression (grouped by median expression) had a worse outcome. Median overall survival in patients with high SNAI2/SLUG expression was 5.7 months versus 11.6 months with low SNAI2/SLUG expression (p = .038). Inversely, patients with high ESR1 expression (grouped by median expression) had an improved median OS with 10.9 months vs. 5.0 months in the low expression group (p = .032). In multivariate analysis, SNAI2/SLUG2 (p = .022) and ESR1 (p = .017) separately were independent prognostic factors for survival.

**Conclusion:**

SNAI2/SLUG is prognostic of patients’ outcome. The strong inverse correlation with ESR1 indicates a significant impact of estrogen receptor pathway regarding these malignant features.

## Background

Lung cancer is the leading cause of death among all malignant diseases worldwide. In the majority of patients (about 70%), the disease is diagnosed in an advanced, non-resectable stage with a very poor outcome. The prognosis is highly associated with the metastatic behavior of the tumor. Metastatic spread is a complex process of molecular and phenotypical changes of tumor cells. In this process the epithelial-mesenchymal transition (EMT) seems to play a crucial role. During EMT cells reduce intercellular adhesions, lose polarity and acquire a fibroblastoid phenotype with high motility and invasive properties [1]. This process is characterized by downregulation of E-cadherin and other epithelial molecules associated with cell adhesion. In parallel, an up-regulation of mesenchymal proteins, like vimentin and an increase of secretion of proteolytic enzymes, like matix-metalloproteinases (MMP), can be observed, contributing to the increase of cell motility, invasiveness and metastatic potential [1,2]. In conclusion, EMT seems to play a key role in the progression of tumors towards invasion and metastasis. Among transcription factors inducing EMT and down regulation of E-cadherin, which represents the hallmark of EMT, the snail family of zing finger transcription factors, like SNAIL (SNAI1) and SLUG (SNAI2) play a prominent role [3]. Overexpression of SNAI2/SLUG can be observed in a variety of different cancers and seems to be associated with poor outcome [4,5]. In particular, SNAI2/SLUG is also a negative prognostic factor for relapse and overall survival in resectable, early stage lung cancer [6,7]. In breast cancer cell lines, Ye et al. [8] could show that SNAI2/SLUG is suppressed by ligand-activation of estrogen receptor α (ERα). Several findings of this study indicate that SNAI2/SLUG is an estradiol- responsive gene and ERα may play an important role in EMT in breast cancer.

To further clarify the role of SNAI2/SLUG in lung cancer and in particular in the advanced setting, this study was conducted to examine the correlation with hormone receptor expression as well as different MMP along with the clinical outcome in Western patients with metastatic NSCLC, enrolled in a randomized first-line chemotherapy trial.

## Methods

### Study population

For mRNA analysis, tumor biopsies of patients with metastatic or advanced NSCLC enrolled in a randomized, multicenter first-line phase II trial and treated with docetaxel and either cisplatin or oxaliplatin [9] were used. These samples were prospectively collected during this study. From a total of 88 randomized patients, tumor samples of 64 patients were available and of those 53 samples qualified for sufficient mRNA extraction and gene expression analysis.

Patients gave informed consent for the study including sample collection and analysis. Approval of the local ethic committees was obtained (leading ethics committee: Landesärztekammer Hessen). Standards of the International Conference on Harmonization World Health Organization (WHO) Good Clinical Practice were followed.

### Sample preparation and RNA extraction

Formalin-fixed paraffin-embedded (FFPE) tissue samples obtained before the start of chemotherapy were collected. From each tumor block, a 5-μm section was stained with hematoxylin–eosin (H&E) and revised by a pathologist and two consecutive 10-μm sections were cut on a standard microtome, placed into individual tubes, and stored at 4°C for ≤1 month until RNA extraction. Fully automated high-throughput RNA extraction has been carried out similar to methods previously published [10] by using a fully automated XTRACT roboter and extraction kits (STRATIFYER Molecular Pathology GmbH, Germany).

### Gene expression analysis using quantitative PCR

Expression of SLUG/SNAI2, MMP2, MMP7, MMP9, estrogen receptor 1 (ESR1) and the normalization (housekeeping) gene CALM2 were assessed by one-step RT-quantitative PCR (qPCR). SuperScript ® III Platinum ® One-Step qRT-PCR System with ROX (Invitrogen, Karlsruhe, Germany) was used according to the manufacturer’s instructions. Experiments were carried out on a Stratagene Mx3005p (Agilent Technologies, Böblingen, Germany) with 30 min at 50°C, 2 min at 95°C followed by 40 cycles of 15 s at 95°C and 30 s at 60°C.

The expression of the genes of interest was calculated by using the ΔC_t_ method. Cycle threshold (C_t_) values, which indicate the (interpolated) number of PCR cycles until the fluorescence reached its threshold, were determined. C_t_ values were normalized by subtracting the C_t_ value of the housekeeping gene (CALM2) from the C_t_ value of the target gene (ΔCT). RNA results were then reported as 40- ΔC_t_ values, which would correlate proportionally to the mRNA expression level of the target gene. For assessment of DNA contamination in RNA preparations, a PAEP gene-specific qPCR without preceding reverse transcription was carried out using the reagents from the SuperScript III® Platinum® One-Step qRT-PCR System with ROX and Taq DNA Polymerase. In samples with a C_t_ value <35, the DNase I treatments were repeated to prevent effects on bispecific PCR assays. Stratagene human QPCR Reference total RNA (Stratagene, Waldbronn, Germany) was used as positive control for RTqPCR and human genomic DNA (Roche Diagnostics, Basel, Switzerland) as positive control for qPCR. All PCR assays were carried out in triplicate, and the mean of triplicates was reported.

### Statistics

The study was explorative. The median expression of genes (SNAI2/SLUG, MMP2, MMP7, MMP9 and ESR1) was used as an objective cut-off to distinguish high from low expression. Associations between gene expression values and clinicopathological data were compared and calculated with chi-square-tests. Progression-free survival (PFS) was measured from the date of assignment until disease progression or death of any cause. Overall survival (OS) was measured from date of assignment until death of any cause. Time-to-event curves were calculated by the Kaplan–Meier method and the log-rank test was applied. The Cox regression model was used for the univariate and multivariate analyses. All P values were two-sided with P values <0.05 indicating statistical significance. Statistical analyses were performed with WinSTAT software (Version 2009.1).

## Results

### SNAI2/SLUG and MMP mRNA expression

SNAI2/SLUG mRNA expression could be evaluated in 49 patients and ranged between 28.01 and 41.70 with a median of 34.09 (ΔC_t_). There was no correlation of SNAI2 expression with clinical characteristics like gender, performance status, stage, histological subtype, number of metastatic sites or treatment, when patients were grouped by the median or the 3rd quartile of SNAI2 expression (Table 1).

**Table 1:** **Patient characteristics**

			SNAI2 median		
Characteristic		Total (*n* = 49)	Low (%)	High (%)	p-value
**Age**					
	Median (Range)	66(39–82)	66(39–82)	66(51–75)	
**Gender**					
	Female	23	11	12	0.778
	Male	26	14	12	
**ECOG PS**					
	Median	1			
	0-1	43	23	20	0.417
	2	6	2	4	
**Histology**					
	Adeno	31	13	18	0.345
	Squamous cell	14	9	5	
	other	4	3	1	
**No. of organs involved**					
	Median				
	≤1	19	10	9	n.s.
	2	18	9	9	n.s.
	≥3	12	6	6	n.s.
**Stage**					
	IIIB / IIIA	2	0	2	0.235
	IV	47	25	22	
**Treatment**					
	Arm A (Cisplatin/Docetaxel)	23	13	10	0.571
	Arm B (Oxaliplatin/Docetaxel)	26	12	14	
**ESR1 status**					
	ESR1 high	25	24	1	<0.0001
	ESR1 low	24	1	23	

ESR1 mRNA expression (*n* = 53) ranged between 28.72 and 39.2 with a median of 35.71. There was no significant association between ESR1 mRNA expression and clinical characteristics, although high ESR1 was slightly more frequently observed in male vs. female patients (*p* = .27), and patients with liver (*p* = .14) and bone metastases (*p* = .16) [11].

MMP2 could be evaluated in 51 patients and ranged between 31.40 and 39.86 (median 36.78). There was no correlation with clinical characteristics. Similar results could be obtained for MMP7 expression (n = 52) which ranged between 30.40 and 42.58 (median 34.98) and MMP9 expression (n = 45) which ranged between 29.91 and 38.75 (median 33.79).

A highly significant inverse correlation could be observed between SNAI2/SLUG and ESR1 mRNA expression (p < 0.0001). In contrast, MMP2 (p = 0.3868), MMP7 (p = 0.3961) and MMP9 mRNA expression (p = 0.366) did not correlate with SNAI2/SLUG (Table 2).

**Table 2:** **Gene expression correlations**

		SNAI2-median		
Characteristic		Low (%)	High (%)	p-value
**ESR1 (n = 49)**				
	high	24	1	<0.0001
	low	1	23	
**MMP2 (n = 48)**				
	high	10	14	0.387
	low	14	10	
**MMP7 (n = 48)**				
	high	14	10	0.396
	low	11	14	
**MMP9 (n = 43)**				
	high	13	9	0.366
	low	9	12	

### Survival analysis

**Figure 1:**
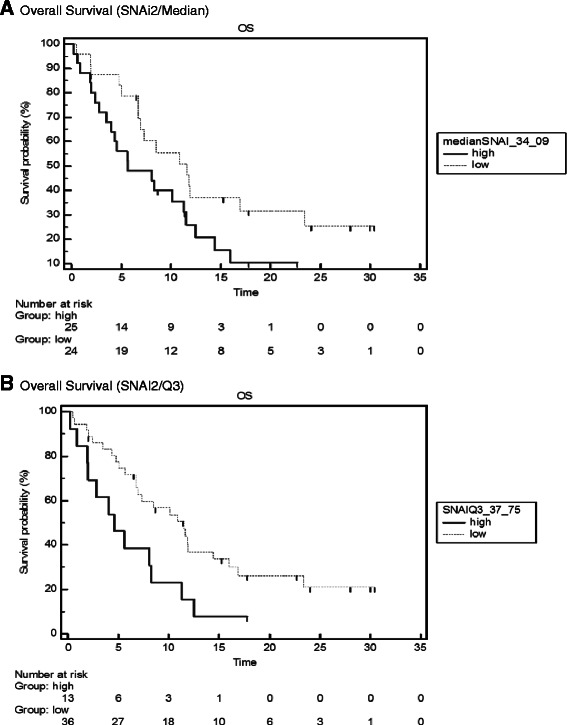
Kaplan-Meier curves for overall survival (OS) grouped by median SLUG/SNAI2 expression **(A)** and by 3rd quartile of SLUG/SNAI2 expression **(B)** for patients with SLUG/SNAI2 high and low tumors. A: median OS (median SLUG/SNAI2 expression): 5.7 vs. 11.6 months, *p* = 0.038, HR 0.52. B: median OS (3rd quartile): 4.6 vs. 11.5 months, *p* = 0.0192, HR 0.45.

In line with previous works of our group [11], ESR1 expression was identified as a marker of favourable outcome in this patient group (n = 53) with a median OS of 10.9 vs. 5.0 months in ESR1 high vs. low patients, respectively (p = .032, HR 0.51). Grouped by the median SNAI2/SLUG expression, OS was 5.7 vs. 11.6 months in the SNAI2/SLUG high vs. low patients (p = .038, HR 0.52) (Figure 1A). When the 3rd quartile of SNAI2/SLUG expression (37.75) was used for classification into high vs. low patients, the differences in OS were even stronger (median OS 4.6 vs 11.5 months, p = .0192, HR 0.45) (Figure 1B).

There was no correlation of different MMPs with survival, median OS was 8.3 vs. 10.5 months for MMP2 high vs. low (p = .431, HR 0.78), 8.3 vs. 9.8 months for MMP7 high vs. low (p = .967, HR 1.01) and 9.8 vs. 10.1 months for MMP9 high vs. low (p = .341, HR 1.39).

### Multivariate analysis

In the multivariate analysis containing the factors gender, age, performance status, histological subtype and SNAI2/SLUG expression, only SNAI2/SLUG expression was significantly associated with OS (p = .022, HR 0.45) (Table 3). In this patient group, ESR1 was also significantly associated with survival in the multivariate analysis (*p* = .015; HR .38), as previously published [11].

**Table 3:** **Multivariate analysis for overall survival**

Covariate	P	HR	95% CI (HR)
**Histology**	0.31	0.64	0.27 - 1.52
**Gender**	0.28	0.68	0.33 – 1.37
**Performance Status**	0.96	0.97	0.35 – 2.67
**Treatment**	0.10	0.54	0.26 – 1.11
**Age**	0.60	0.80	0.34 – 1.85
**SNAI2 expression**	0.02	0.45	0.22 – 0.90

## Discussion

EMT has emerged as a critical phenomenon in the carcinogenesis. There is growing evidence that EMT occurs also during lung cancer development. In a series of adenocarcinomas and squamous cell carcinomas, Prudkin and co-workers [12] showed that EMT phenotype was found in most of the lung tumors in contrast to dysplastic lesions or adjacent bronchial epithelium. Shintani et al. [13] compared tissue specimens of patients with NSCLC who received preoperative radiochemotherapy. They observed an EMT marker expression increase in 40% of patients and this correlated with poor outcome (disease free-survival).

In stage I NSCLC, EMT markers such as Twist and SNAI2/SLUG were associated with a worse overall survival and recurrence-free survival [7]. In resected adenocarcinoma of the lung, Shih et al. [6] could confirm the negative prognostic effect of SNAI2/SLUG expression measured by mRNA on survival and relapse. They also observed an increase of MMP2-mRNA expression in SNAI2/SLUG overexpressing tumors.

EMT in lung cancer is triggered by multiple intrinsic and extrinsic factors. One of the most important characterized EMT-inducing oncogenic changes is the K-RAS mutation [14,15]. Additionally, TGF-β and hypoxia inducing factor-2α (HIF-2α) are known EMT inducing intrinsic factors. Among extrinsic factors, hypoxia and inflammatory tumor microenvironment have to be mentioned.

Interestingly, tobacco smoking induced EMT through HDAC –mediated down regulation of E-cadherin via up regulation of SNAI2/SLUG [16].

In our study, we could confirm the negative prognostic effect of SNAI2/SLUG expression in advanced lung cancer, indicating the prognostic effect of EMT. It has to be noticed that in comparison to previously published data on early stage operable patients, our cohort consisted of advanced or metastatic stages, so this phenomenon seems not only be restricted to early stage lung cancer.

In contrast to findings of Shih et al. and others groups [17], we could not observe a correlation of SNAI2/SLUG and MMP 2 in our cohort. Probably this could be due to the limited patient number in our series. However, a difference in early stage and metastatic cancers has to be considered as a potential explanation for this observation.

One important finding of our study was the significant inverse correlation of SNAI2/SLUG and ESR1. This in line with results reported from breast cancer, where SNAI2/SLUG expression was evaluated in different ER-positive and ER negative cell lines [18]. Estrogen receptor-α directly repressed transcription of SNAI2/SLUG by the formation of a complex of ligand-activated estrogen receptor-α, histone deacetylase 1 and nuclear receptor corepressor (N-CoR) [8].

This phenomenon can also be observed in ovarian cancer. Park et al. [19] could show, that E-Cadherin suppression and SNAI2/SLUG expression is mediated by estradiol and estrogen receptor alpha in ovarian cancer cell lines.

Our findings fit into the consistent overall picture that a distinct subgroup of non-small cell lung cancer (ESR1 high expression tumors) has certain similarity and analogy to breast cancer, based on several epidemiologic and observational studies.

First, in a previous study [11] we could show that ESR1 is an independent prognostic factor in metastatic NSCLC similar to breast cancer.

Second, the metastatic pattern/ and behavior of ESR1 positive lung cancer is similar to breast cancer, where bone metastases are associated with estrogen receptor positivity [11].

Third, SNAI2/SLUG is significantly inversely correlated with ESR1 expression and prognostic in analogy to breast cancer.

Furthermore, the strong inverse correlation of SNAI2/SLUG with ESR1 underlines and validates the prognostic relevance of ESR1 in lung cancer. As SNAI2/SLUG is one of the key factors for E-cadherin suppression and for EMT, which represents a more aggressive phenotype of cancer, the results seem reasonable.

With our data we cannot provide a proof that SNAI2/SLUG expression is directly triggered by the estrogen pathway and we cannot rule out that the strong inverse correlation of SNAI2/SLUG and ESR1 is determined by an independent pathway. However, the analogy to breast and ovarian cancer suggests that SNAI2/SLUG is an ER responsive gene in lung cancer as well.

Our results would have two implications in NSCLC patients. First, SNAI2/SLUG expression adds to the prognostic factors known in NSCLC, making it meaningful to stratify according to SNAI2/SLUG in future clinical trials. Additionally, it helps to identify patients with poor prognosis, who may be candidates for more aggressive therapies in the future. Second, based on the strong inverse correlation with ESR1 expression, SNAI2/SLUG expression and EMT in general should be studied in response to antihormonal treatment in vitro and in vivo.

Taken together, our data confirm that, as for breast cancer, ESR1 expression in lung cancer is associated with the lower levels of EMT Markers. Therefore, the results warrant further evaluation of antihormonal treatment in a subgroup of patients with lung cancer (ESR1 high lung cancer) in analogy to ER/PR positive breast cancer.

## Conclusion

SNAI2/SLUG is prognostic of patients’ outcome. The strong inverse correlation with ESR1 indicates a significant impact of estrogen receptor pathway regarding these malignant features.
